# Morphological Variation between Hatchery Bred and Wild Caught *Anabas testudineus* in Malaysia

**DOI:** 10.21315/tlsr2022.33.1.12

**Published:** 2022-03-31

**Authors:** Okomoda Victor Tosin, Ahmad Azfar Mohamed, Nur Asma Ariffin, Ahmed Jalal Khan Chowdhury, Abol-Munafi Ambok Bolong

**Affiliations:** 1 Department of Fisheries and Aquaculture, College of Forestry and Fisheries, University of Agriculture, P.M.B. 2373 Makurdi, Nigeria; 2 Institute of Tropical Aquaculture and Fisheries Research (AKUATROP), Universiti Malaysia Terengganu, 21030 Kuala Nerus, Terengganu, Malaysia; 3 Department of Marine Science, Kulliyyah of Science, International Islamic Universiti Malaysia, 25200 Kuantan, Malaysia; 4 Faculty of Food Science and Fisheries, Universiti Malaysia Terengganu, 21030 Kuala Nerus, Terengganu, Malaysia

**Keywords:** Climbing Perch, Morphometric Parameters, PCA Analysis, Environmental Plasticity, Ikan Puyu, Parameter Morfometrik, Analisis PCA, Keplastikan Persekitaran

## Abstract

This study was designed to characterise the cultured and wild populations of *Anabas testudineus* in Malaysia using morphological parameters. Fish samples from the East and West coast of the country were obtained from fishermen (wild samples) and well-recognised climbing perch farmers in Kedah, Kelantan, Johor and Selangor. The Truss network method was applied to obtain necessary data and analysed to examine phenotypic variation between the cultured and wild stocks. Results obtained suggest that each hatchery population belonged to a distinct stock as revealed by their separate clustering into individual unique groups. However, an extensive overlap was observed in the wild population suggesting similarity of origin. The most important morphological parameters for the discrimination of the two populations are the homologous landmark B (i.e., snout to insertion of the pelvic fin) and C (i.e., above the eye to insertion of the pelvic fin). Genetic characterisation of the *A. testudineus* is needed to complement the findings of this study and establish a baseline for the development of a selective breeding programme for the fish species in Malaysia.

## Abstract (fr)

Kajian ini dijalankan untuk mencirikan populasi *Anabas testudineus* yang dikultur dan liar di Malaysia menggunakan parameter morfologi. Sampel ikan dari pantai timur dan barat negara ini diperoleh daripada nelayan (sampel liar) dan penternak ikan yang diiktiraf di Kedah, Kelantan, Johor dan Selangor. Kaedah rangkaian Truss digunakan untuk mendapatkan data yang diperlukan dan dianalisis untuk memeriksa variasi fenotip antara stok kultur dan liar. Keputusan yang diperoleh menunjukkan bahawa setiap populasi penetasan tergolong dalam stok yang berbeza seperti yang didedahkan oleh kelompok berasingan ke dalam kumpulan unik individu. Walau bagaimanapun, pertindihan yang luas diperhatikan dalam populasi liar yang mencadangkan persamaan asal. Parameter morfologi yang paling penting untuk diskriminasi kedua-dua populasi ialah mercu tanda homolog B (iaitu, snout untuk memasukkan sirip pelvis) dan C (iaitu, di atas mata untuk memasukkan sirip pelvis). Pencirian genetik *A. testudineus* diperlukan untuk melengkapkan penemuan kajian ini dan mewujudkan asas untuk pembangunan program pembiakan terpilih untuk spesies ikan di Malaysia.

**Highlight**

- Based on the unique individual clustering of the hatchery population, it was concluded that each hatchery population belong to different stocks.
- The wild population however seem to come from a similar origin due to an extensive overlap noticeable.
- The measurement of the snout to the insertion of the pelvic fin and above the eye to insertion of the pelvic fin were the most important parameters that can be used to discriminate the two populations.

## INTRODUCTION

The climbing perch *Anabas testudineus* is one of the economically important and highly valued fish species around the world (Ambak *et al*. 2010; Chaturvedi *et al*. 2015). It is widely distributed in freshwaters, brackish waters, and estuaries of several countries in Asia (Zalina *et al*. 2012; Bungas *et al*. 2013). The popularity of this species is predicated on its hardiness; hence, it can tolerate unfavourable water conditions, like high turbidity and variations in salinity up to 12 ppt (Hitchcock 2008; Chotipuntu & Avakul 2011). This is because the fish is an obligatory air-breathing species that is capable of surviving for prolonged hours outside the water (Hughes *et al*. 1986). Consequently, *A. testudineus* has prolonged freshness even when out of water for a long time and is traditionally esteemed for its fine flavour (Rahman *et al*. 2013).

The non-pigmented and transparent nature of the egg has also made this fish an important animal model for diverse biological studies due to the ease of observation of early life development (Hassan *et al*. 2018). Several fundamental studies earlier conducted have led to the domestication, breeding, and aquaculture production of the *A. testudineus* (Atal *et al*. 2009; Cacot *et al*. 2009; Kohinoor *et al*. 2013). However, there is a paucity of information about morphological variation between domesticated hatchery-bred stocks and their wild-caught counterparts. Such studies in conjunction with genetic characterisation can be the basis upon which a selective breeding programme could be conducted aimed at improving the performance characteristics of fish through the cross-breeding of strains (Solomon *et al*. 2015).

According to Normala *et al*. (2017), conventional morphological methods continue to have an important role in stock identification even despite the development of advanced techniques that can directly examine biochemical or genetic variations in the fish. The criticism about traditional morphometric measurements being contradictory and generating ambiguous results (Garrido-Ramos *et al*. 1997; Doukakis *et al*. 2000) has given rise to the development of geometric landmark-based techniques. Hence, this is currently the most rigorous morphometric technique used around the world (Barriga-Sosa *et al*. 2004; Pinheiro *et al*. 2005; Bagherian & Rahmani 2009). Therefore, the current study which seeks to investigate the landmark-based morphological variations between hatchery bred and the wild populations of *A. testudineus* in Malaysia represents the first step towards developing a selective breeding programme for the species.

## MATERIALS AND METHODS

### Study Area and Sample Collection

The samples of *A. testudineus* were collected at 12 different sites in four states around Peninsular Malaysia (Kedah, Kelantan, Johor and Selangor) as shown in Table 1. The wild samples were collected from contracted fishermen from the three states and were caught using gillnet and angling. For the hatchery samples, collections were made from hatcheries listed by the Department of Fisheries (DOF) directory as the main captive producer of *A. testudineus* in Malaysia. It should be noted that the standard operating procedures of the different hatcheries differ in terms of culture and care for the fish under captive conditions. However, this was not thought to be a source of bias for delineating the morphometric parameters of the fish.

All the collected samples were placed on ice and transported to the bioscience laboratory at the Faculty of Food Science and Fisheries for further morphological analyses. The fish sample’s identity was confirmed using the identification key given by Ambak *et al*. (2010). Thereafter, they were tagged for morphological analysis. The experimental protocols for this study were approved by the Universiti Malaysia Terengganu committee on research. All methods used in this study involving the care and use of animals were following international, national and institutional guidelines (Protocol QL638.99.N6).

### Morphological Discrimination of ***A. testudineus*** using Truss Network

**Figure 1:**
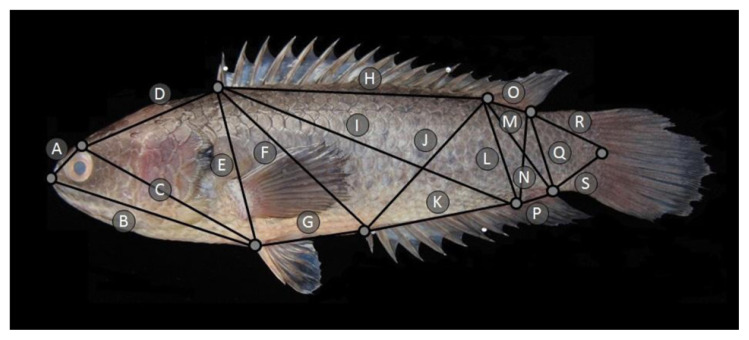
Illustration of *A. testudineus*, showing 10 locations of the homologous landmark for constructing the truss network measurement based on morphological features.

The morphological measurements were conducted based on a truss network anchored on 10 homologous landmarks as shown in Fig. 1. This resulted in 19 linear measurements as detailed in Table 2. Pictures of the *A. testudineus* were captured using a Sony camera (Cyber-shot 16.2MP Model number: DSC-TX10 50i, Japan). The photos were then measured using the NIS element Basic Research software.

### Morphological Data Analysis

The original measurements of the Truss network were firstly standardised to remove the size effect from the data set (Murta *et al*. 2008; Jaferian *et al*. 2010). Hence, to normalise the individuals in a sample to a single arbitrary size, common to all samples, but maintains the individual variation (Sen *et al*. 2011), the allometric formula by Elliott *et al*. (1995) was used as shown:


$${\text{M}}_{\text{adj}}=\text{M }{\left(\text{Ls}/{\text{L}}_{\text{o}}\right)}^{\text{b}}$$

Where

M = Observed character measurement;
M_adj_ = size-adjusted measurement;
L_o_ = Standard length of the fish;
Ls = Overall mean of the TL for all the progenies;
b = Estimated for each character from the collected data as the slope of the regression of log M on log L_o_, using all fish of all the progenies.

The data obtained from the Truss network technique were analysed by the multivariate analysis of discriminant function analysis (DFA) using Statistical Package for Social Science (SPSS) version 16.0 software for windows. The relative importance of discriminant variables (functions) is determined on some basic measures namely: the relative percentage of the Eigenvalue/the percent of variance existing in the discriminating values and the associated canonical correlation. The sample centroids graph was also done using the SPSS software to see the separation on the biplot and determine the morphological character that can be used to separate the fish samples into distinct groups.

## RESULTS

**Figure 2:**
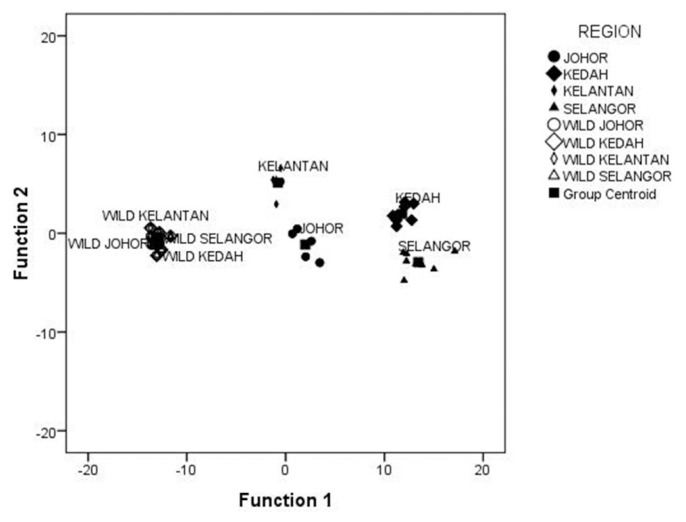
Scatter plot of DFA scored based on morphometric characters of hatchery and wild *A. testudineus* populations.

The result for the DFA for the transformed homologous landmark distances of *A. testudineus* is presented in Table 3. The Eigenvalue of the first four principal components was selected, as they were more than 1, following the recommendations of Kaiser (1961). Results obtained revealed that the first principal component (PC1) accounted for 91.6% of the total variance with only positive coefficients. Principal component 2 (PC2) had positive and negative coefficients and counted for 4.3% of the total variance in the samples. Similarly, PC3 and PC4 accounted for 2.6% and 1.4% of the total variation, respectively. In total, all four principal components accounted for 99.8% of the variance observed for *A. testudineus* from cultured and wild populations. Canonical correlations in the four principal components were also high ranging from 0.996 (PC1) to 0.815 (PC 4). In general, the most influential variables of the principal components include the homologous landmark B (i.e., the snout to insertion of the pelvic fin) and C (i.e., above the eye to insertion of the pelvic fin). The means of canonical variances scores and the scattered plot are presented in Table 4 and Fig. 2. The result obtained showed a cluster of all wild populations into a single group. However, the hatchery-bred population was distinctly separated from one another without any noticeable overlap between them or the wild population.

## DISCUSSION

Stock discrimination is fundamental to fisheries and hatchery management (Begg & Waldman 1999). This is because of its importance in designing and managing a productive breeding programme (Wedemeyer 2001). Fish stocks are identified based on differences in the characteristic among different groups with similar life histories (Hossain *et al*. 2010). Generally, fishes are known to be the most susceptible to environmentally induced morphological variations among vertebrates (Allendorf *et al*. 1987; Wimberger 1992; Solomon *et al*. 2015). Just like many other studies, the morphological characteristic has proven useful in the discrimination of hatchery bred and the wild population of *A. testudineus* from different parts of Malaysia. This is based on the observation of a clear separation among the hatchery population and distinct from the wild stock. Hence, suggesting that each population in the cultured stock belongs to different stocks while all the wild population belongs to a single distinct stock.

Similar to the finding of this study, Swain *et al*. (1991) had shown that wild and hatchery Coho salmon could be distinguished using a Truss network system. Phenotypic differences in *Clarias gariepinus* and endangered *Tor putitora* have also been reported to be high for different environments (i.e., culture and wild) in the study reported by Solomon *et al*. (2015) and Patiyal *et al*. (2014), respectively. The variation observed between wild and hatchery populations has been suggested to be due to different rearing conditions in the environment rather than genetic differences between the stocks (Swain *et al*. 1991). Husbandry conditions in the wild and captive environments are far apart in many ways. The hatchery provides almost everything to ensure better survival of the reared fish in a restricted environment while the natural environment conditions are uncertain and survival of the fittest is championed by natural selection (Wedemeyer 2001; Sedwick 1995). Many researchers have highlighted the role of environmental conditions such as food abundance and temperature as an important cause of fish morphological plasticity. These parameters greatly differ for wild and hatchery environments. Hence, despite the hatchery population being a fragment of the wild population, the various process of domestication, artificial selection adopted in hatchery over the years, and husbandry methods used may have impacted the morphological parameters significantly (Blanchet *et al*. 2008).

Ahmad (2015) had also opined that domestication and adaptation of fish to the various hatchery conditions are potential courses of observed morphological deviation of hatchery and wild stock. It could then be right to hypothesise that the different husbandry practices used in the different hatcheries in this study may have also resulted in the emergence of distinct stocks for the different stocks of *A. testudineus* studied. Another school of thought has also suggested that morphological variation in culture fishes could have resulted from gene pollution of the original domesticated stock occasioned by several years of inbreeding and accidental hybridisation with closely related species (Olufeagba *et al*. 2002; El-Serafy *et al*. 2007; Solomon *et al*. 2015; Okomoda *et al*. 2018; Okomoda 2018). However, this can only be correctly inferred by genetic characterisation. This study observed that the most important morphological parameters for the discrimination of both cultured and wild populations were related to the head. An earlier study by Leslie and Grant (1990), Schweigert (1990), and Haddon and Willis (1995) have revealed that morphological measurement of the head and body depth are the most important characters of discrimination in samples of *Lophius vomerinus*, *Clupea pallasi*, and *Hoplostethus atlanticus*, respectively. In African catfish too, Turan *et al*. (2005) and Solomon *et al*. (2015) had reported that *C. gariepinus* samples from the wild and the cultured environment can be discriminated against using head-related morphometric parameters.

Although morphometric differences among stocks have been linked to differences in geographical and ancestral origins (Hossain *et al*. 2010), our findings for the wild population suggest they all fit into a single distinct group. Contrary to this finding, the reports of Turan *et al*. (2004) for wild populations of *Liza abu* from the Orontes, Euphrates, and Tigris rivers in Turkey show the fish were differentiated into distinct groups using morphometric parameters. Wedemeyer (2001) had stated that natural environmental influences cause a random process of mutation, genetic drift, and gene flow resulting in changes in wild population morphology. The similarity of wild stock in the current study may be suggestive of similarity in the environmental factors of the wild in the different states sampled or the ancestral origins of the *A. testudineus*.

Since each hatchery stock described in this study is individually distinct morphometrically, mating broodstock from the different hatchery stock or with wild lineages could theoretically help to produce offspring with better performance (Aung *et al*. 2010). The selection of wild population as a supplement to hatchery population has been used in some previous selective breeding programmes aimed at improving the fitness of hatchery bred fishes especially in the area of disease-resistant (Kapuscinski *et al*. 1996; Uraiwan *et al*. 2007). This could be investigated in future research for the *A. testudineus* samples reported in this study. However, it should be noted that mating broodstock from a distinct population might not translate into an improvement of the performance of cultured fishes; but, it can increase variation within the hatchery population which could be important for future breeding programmes (Tallmon *et al*. 2004).

## CONCLUSION

Through the morphological data collected, the study concluded that each hatchery population belonged to a distinct stock, however, the wild population is likely from a similar origin as indicated by the extensive overlap noticeable in the biplot. Despite the findings of this study, the genetic characterisation of the fish (i.e., *A. testudineus*) must be conducted to validate the reports of this study.

## Figures and Tables

**Table 1:** Sampling sites coordinate and sample size of *A. testudineus* collected from both hatcheries and wild population.

Region	Sampling site	Latitude	Longitude	Sample size (N)	Remark
Kedah	Alor Setar	6°06′14″	100°21′02″	9	Wild
	Kuala Muda	5°35′26″	100°22′24″	11	Wild
	Sungai Petani	5°38′27″	100°29′15″	12	Wild
	Serdang	5°12′39″	100°36′58″	35	Hatchery
Kelantan	Tumpat	5°53′23″	102°28′52″	10	Wild
	Kota Bharu	6°07′15″	102°14′36″	12	Wild
	Tok Bali	6°12′10″	102°09′29″	8	Wild
	Pasir Puteh	5°53′57″	102°20′19″	35	Hatchery
Johor	Segamat	2°30′31″	102°48′53″	9	Wild
	Tangkak	2°17′01″	102°33′02″	7	Wild
	Parit Jawa	1°56′32″	102°39′50″	15	Wild
	Batu Pahat	1°42′33″	103°09′01″	30	Hatchery
Selangor	Tg. Karang	3°28′25″	101°12′39″	12	Wild
	Sungai Buloh	3°15′20″	101°18′17″	7	Wild
	Kuala Selangor	3°19′59″	101°13′52″	14	Wild
	Rawang	3°18′31″	101°32′50″	40	Hatchery

**Table 2:** Morphometric distances measured between landmark points of *A. testudineus*.

Homologous Landmark	Character description
A	The snout to above the eye
B	The snout to insertion of the pelvic fin
C	Above the eye to insertion of the pelvic fin
D	Above eye to the origin of the dorsal fin
E	Origin of the dorsal fin to insertion of the pelvic fin
F	Origin of the dorsal fin to the origin of the anal fin
G	Insertion of the pelvic fin to the origin of the anal fin
H	Origin of the dorsal fin to the origin of the dorsal soft rays
I	Origin of the dorsal fin to the origin of the anal soft rays
J	Origin of the anal fin to the origin of the dorsal soft rays
K	Origin of the anal fin to the origin of the anal soft rays
L	Origin of the dorsal soft rays to origin of the anal soft rays
M	Origin of the dorsal soft rays to end of the anal soft rays
N	Origin of the anal soft rays to end of the dorsal soft rays
O	Origin of the dorsal soft rays to end of the dorsal soft rays
P	Origin of the anal soft rays to end of the anal soft rays
Q	End of the dorsal soft rays to end of the anal soft rays
R	End of the dorsal soft rays to the caudal fin
S	End of the anal soft rays to the caudal fin

**Table 3:** The summary of eigenvalue, canonical correlation and standardised canonical discriminant function coefficients scored based on morphometric characters of hatchery and wild *A. testudineus* populations.

Variables	Function			

	1	2	3	4
A	0.069	0.168	0.531	−0.304
B	0.356	−0.102	−0.074	−0.162
C	0.351	−0.336	−0.094	−0.031
D	0.206	0.012	0.059	0.031
E	0.093	−0.162	0.180	−0.175
F	0.165	−0.198	−0.010	−0.221
G	0.166	−0.022	0.028	0.116
H	0.274	−0.066	−0.083	−0.184
I	0.216	−0.062	0.029	−0.297
J	0.109	−0.009	0.176	−0.307
K	0.102	0.063	−0.039	−0.407
L	0.043	−0.004	0.104	−0.291
M	0.095	0.014	0.153	−0.048
N	0.098	0.010	0.121	−0.213
O	0.051	0.020	−0.070	−0.085
P	0.039	0.034	0.105	0.104
Q	0.069	−0.160	−0.070	−0.009
R	0.100	0.048	−0.213	0.024
S	0.103	0.081	−0.238	0.017
Eigenvalue	134.137	6.299	3.781	1.982
% of variance	91.6	4.3	2.6	1.4
Cumulative % variance	91.6	95.9	98.5	99.8
Canonical correlation	0.996	0.929	0.889	0.815

*Note*: Pooled within-groups correlations between discriminating variables and standardised canonical discriminant functions. Variables are ordered by the absolute size of correlation within function*. Largest absolute correlation between each variable and any discriminant function.

**Table 4:** Functions at group centroids/means of canonical variances scores from morphological differences between hatchery and wild populations of *A. testudineus*.

Region	Function			

	1	2	3	4
Johor	1.986	−1.147	0.110	3.635
Kedah	11.792	1.973	2.897	−0.453
Kelantan	−0.799	5.123	−3.210	0.052
Selangor	13.432	−2.920	−1.830	−0.877
Wild Johor	−13.213	−0.816	0.532	−0.397
Wild Kedah	−12.775	−0.766	0.293	−0.332
Wild Kelantan	−13.043	−0.369	0.371	−0.538
Wild Selangor	−12.901	−1.125	0.544	−0.681

*Note*: Unstandardised canonical discriminant functions evaluated at group means.
